# A pro-inflammatory mediator USP11 enhances the stability of p53 and inhibits KLF2 in intracerebral hemorrhage

**DOI:** 10.1016/j.omtm.2021.01.015

**Published:** 2021-02-04

**Authors:** Xiuqing Zhang, Tiejun Liu, Shijun Xu, Peng Gao, Wei Dong, Weiran Liu, Ming Gao, Lihua Song, Lusha Cui, Xiaoliu Dong

**Affiliations:** 1 Department of Neurorehabilitation, Tangshan People’s Hospital, Tangshan 063000, P.R. China; 2 Department of Anesthesiology, North China University of Science and Technology Affiliated Hospital, Tangshan 063000, P.R. China; 3 Department of Radiation Oncology, Tangshan People’s Hospital, Tangshan 063000, P.R. China; 4 Department of Neurosurgery, Tangshan People’s Hospital, Tangshan 063000, P.R. China; 5 Department of Five Ward, Kailuan Mental Health Center, Tangshan 063000, P.R. China

**Keywords:** intracerebral hemorrhage, ubiquitin-specific protease 11, p53, Kruppel-like factor 2, NF-κB pathway, neuroinflammation

## Abstract

Microglial cell activation and neuroinflammation after intracerebral hemorrhage (ICH) lead to secondary brain damage. Ubiquitin-specific protease 11 (USP11) has been correlated with ICH-induced neuron apoptosis. This study aims to explore the molecular mechanism of USP11 regulating neuroinflammation in ICH. First, an ICH rat model was developed by intracranial administration of collagenase. Silencing USP11 was found to alleviate nerve injury in rats with ICH-like symptoms. Then, through loss- and gain-of-function assays, USP11 knockdown was revealed to alleviate ICH-induced symptoms, corresponding to reduced modified neurological severity scores (mNSS) value, brain water content, blood-brain barrier permeability, neuron apoptosis, microglial cell activation, neutrophil infiltration, and inflammatory factor secretion. It was subsequently shown in microglial cells that USP11 stabilized p53 by deubiquitination and p53 targeted the Kruppel-like factor 2 (KLF2) promoter to repress KLF2 transcription, thereby activating the nuclear factor κB (NF-κB) pathway. Further, rescue experiments were conducted *in vivo* to validate the function of the USP11/p53/KLF2/NF-κB axis in ICH-induced inflammation, which confirmed that USP11 silencing blocked the release of pro-inflammatory cytokines following ICH by downregulating p53, thus protecting against neurological impairment. Hence silencing USP11 may be a novel anti-inflammatory method for ICH treatment.

## Graphical Abstract

**Figure:**
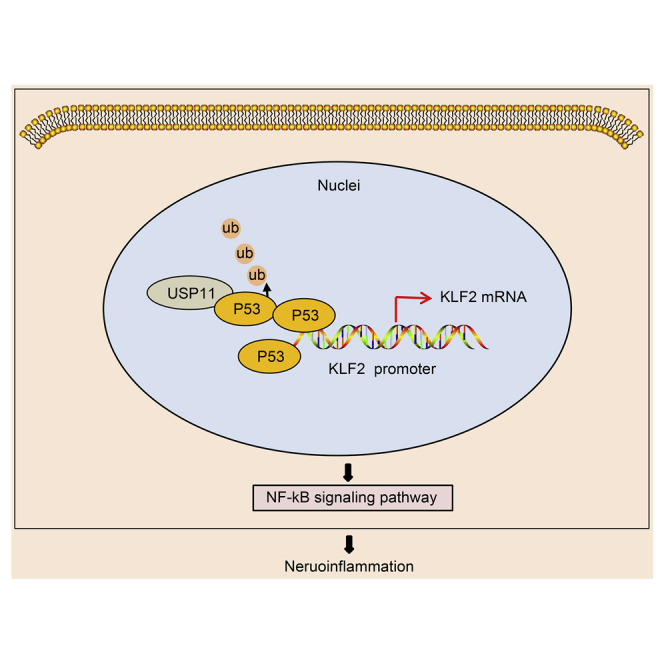

## Teaser

This study demonstrates that silencing USP11 blocks the release of pro-inflammatory cytokines following intracerebral hemorrhage by inhibiting the activation of the NF-κB pathway through the P53/KLF2 axis, thus protecting against neurological impairment. Hence silencing USP11 may be a novel anti-inflammatory method for ICH treatment.

## Introduction

Intracerebral hemorrhage (ICH), or cerebral bleed, represents a devastating subtype of stroke with significantly high morbidity and mortality.^1^ The treatment outcomes of ICH are dependent on the site, mass effect, and intracranial pressure induced by hematoma and also are affected by cerebral edema secondary to perihematomal neurotoxicity or inflammatory damage and complications following delayed neurological impairment.^2^ Based on improved understanding of ICH pathogenesis, pharmaceutical and molecule-targeted therapy have been demonstrated to prevent the hemorrhage, relieve the impact of cerebral edema, and improve the survival.^3^^,^^4^ Notably, microglial activation is an important contributor to neuroinflammation responding to ICH, and hence therapeutic options aimed at mediating microglial cell function may mitigate ICH damage.^5^ Therefore, pathophysiological investigation on the molecular mechanism related to microglial activation-induced neuroinflammation in ICH is urgent and beneficial for developing potential therapeutic interventions.^6^

Ubiquitin-specific protease 11 (USP11), a deubiquitylase mainly localized in the nucleus, belongs to the USP family.^7^ Previous research has identified the correlation of USP11 with the neuronal apoptosis following ICH,^8^ but an underlying molecular mechanism remains unclear. Although the function of USP11 has been reported in signaling transduction,^9^ chromatin reorganization,^10^ and cancer cell apoptosis^11^ through deubiquitination and stabilization of key genes related to those processes, little is known about its role in the central nervous system and neuroinflammation.

p53 is a well-studied tumor suppressor.^12^ Both in cancer and normal somatic cells, p53 activity is regulated by ubiquitination-induced degradation and deubiquitination-induced stabilization.^13^ Members from the USP family, such as USP2a,^14^ USP7,^15^ and USP10,^16^ have been reported to affect the p53 pathway either by targeting ubiquitin ligase or by deubiquitinating p53 itself. Impacts of USP11 on the p53 pathway have been observed in response to tumor necrosis factor alpha (TNF-α) stimulation^17^ and DNA damage.^18^ Despite that earlier research studies of p53 have focused on the transcriptional activities, p53 can downregulate the expression of downstream genes,^19^ one of which is Kruppel-like factor 2 (KLF2).^20^ It has been demonstrated that p53 inhibited KLF2 by binding to its promoter region.^21^ KLF2 is involved in a variety of physiological and pathological processes, including adipogenesis,^22^ embryonic erythropoiesis,^23^ and subarachnoid hemorrhage.^24^

Nuclear factor κB (NF-κB) is a protein that controls many processes, including transcription of DNA, cytokine production, and cell survival.^25^ NF-κB is closely regulated by KLF2 in inflammation,^26^ monocytic activation,^27^ and neutrophil accumulation.^28^ Studies have demonstrated that the activation of NF-κB in the microglial cells and macrophages leads to secretion of proinflammatory cytokines and brain damage after ICH.^29^^,^^30^ Another study also revealed that NF-κB activation may promote inflammation triggered by ICH.^31^ On the basis of aforementioned evidence, we reasonably proposed a modulatory axis by which USP11 may affect neurological impairment and inflammation damage in ICH. Herein, our study focused on the roles and interaction of USP11, p53, KLF2, and NF-κB in neurological function and inflammatory response in the ICH rat model, so as to find neuroprotective and anti-inflammatory targets against ICH.

## Results

### USP11 silencing alleviates nerve injury of rats with ICH-like symptoms

**Figure 1:**
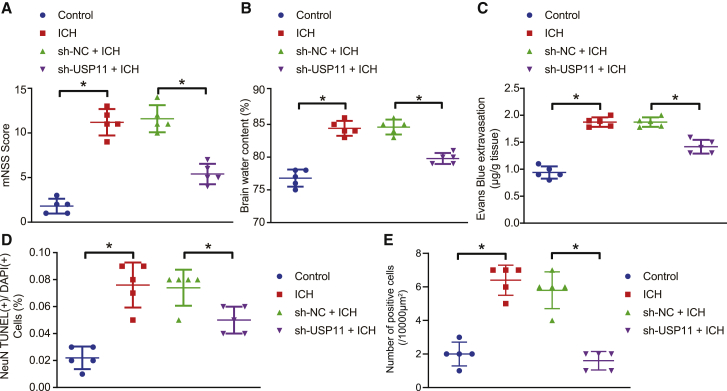
Silencing USP11 protects against nerve injury in the ICH rat model (A) mNSS after 72 h of ICH induction and lentivirus infection. (B) Water content measured by wet-dry weight method in rat brain after 72 h of ICH induction. (C) BBB permeability measured by Evans blue staining in rat brain tissues after 72 h of ICH induction and lentivirus infection. (D) Quantitative analysis of neuron apoptosis by TUNEL and NeuN staining in the brain tissues after 72 h of ICH induction and lentivirus infection. (E) Immunohistochemical staining for USP11 expression in the brain tissues after 72 h of ICH induction and lentivirus infection. ∗p < 0.05. Measurement data are presented as mean ± standard deviation. Comparisons among multiple groups were performed by one-way ANOVA, followed by Dunnett’s post hoc test. n = 5.

To validate the role of USP11 in ICH, we constructed an ICH rat model by intracranial injection with type IV collagenase and performed USP11 loss-of-function experiment. The nerve injury following USP11 silencing was assessed by a modified neurological severity scores (mNSS) system. Compared with the sham-operated rats, the mNSS value was raised in the rats that received intracranial injection with type IV collagenase, whereas this value was reduced after USP11 silencing (Figure 1A). The brain water content in the rats that received intracranial injection with type IV collagenase was higher than that in the sham-operated rats, whereas the brain water content was reduced when USP11 was knocked down (Figure 1B). According to the results obtained from Evans blue staining, blood-brain barrier (BBB) permeability was increased in the rats that received intracranial injection with type IV collagenase relative to the sham-operated rats. However, downregulation of USP11 reduced the BBB permeability (Figure 1C). TUNEL and neuronal nuclear antigen (NeuN) co-staining was performed to monitor the neuron apoptosis in the brain tissues. Neuronal apoptosis rate in the brain tissues was increased considerably in the rats that received intracranial injection with type IV collagenase, which was suppressed by USP11 knockdown (Figure 1D). Furthermore, levels of USP11 in the brain tissues were detected by immunohistochemical staining. USP11 was highly expressed in the rats that developed ICH-like symptoms but reduced after infection with lentivirus expressing sh-USP11 (Figure 1E). From the results above, it was concluded that USP11 loss of function relieved nerve injury in rats with ICH-like symptoms.

### USP11 silencing inhibits the ICH-induced activation of microglial cells and inflammatory damage in brain tissues

**Figure 2:**
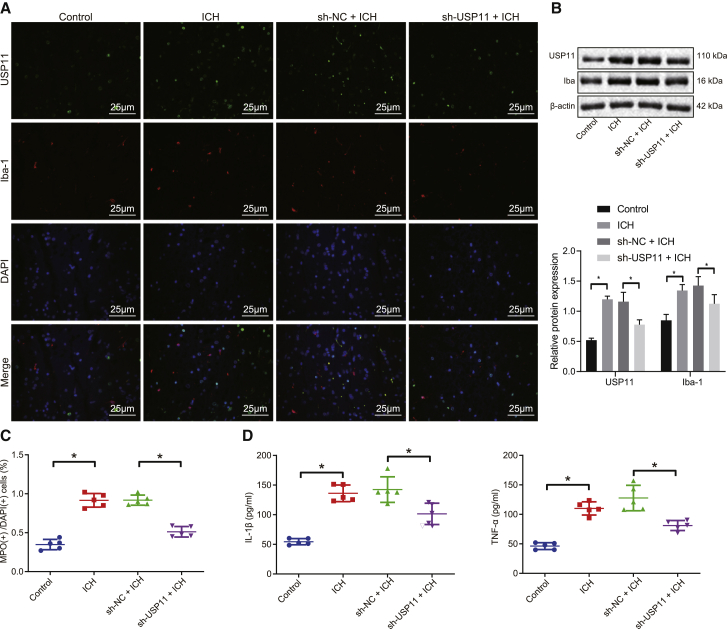
Loss of USP11 inhibits microglial cell activation, neutrophil infiltration, and inflammation in brain tissues of rats with ICH-like symptoms (A) Representative images showing co-localization of USP11 (green), Iba-1 (red), and DAPI (blue) in the brain tissues detected by immunofluorescence (original magnification 400×). (B) USP11 and Iba-1 protein levels in the rat brain tissue measured by western blot assay. (C) Quantitative analysis of immunofluorescence staining for neutrophil activation marker MPO and DAPI in the rat brain tissues. (D) Contents of inflammatory factors TNF-α and IL-1β in the rat brain tissues determined by ELISA. ∗p < 0.05. Measurement data are presented as mean ± standard deviation. Comparisons among multiple groups were performed by one-way ANOVA, followed by Dunnett’s post hoc test. n = 5.

Next, this study investigated the colocalization of USP11 and ionized calcium-binding adaptor molecule 1 (Iba-1), a microglial marker gene, in the brain tissues of rats by conducting immunofluorescence assay. As shown in Figure 2A, the fluorescence intensities of USP11 and Iba-1 were boosted in the rats with ICH-like symptoms, and Iba-1 expression was reduced in response to USP11 downregulation. Western blot assays provided consistent increases in USP11 and Iba-1 protein levels in the rats with ICH-like symptoms and reduction in Iba-1 protein level in response to silencing of USP11 (Figure 2B). Furthermore, the relationship between ICH-induced inflammation and USP11 was investigated by assessing neutrophil infiltration and the production of inflammatory factors. The neutrophil infiltration was observed in rats with ICH-like symptoms, which was eliminated by inhibiting USP11 (Figure 2C). The enzyme-linked immunosorbent assay (ELISA) results suggested the increased production of TNF-α and interleukin-1β (IL-1β) in the brain tissues of rats with ICH-like symptoms, while USP11 silencing reduced their levels (Figure 2D). Taken together, it could be concluded that USP11 knockdown blocked microglial cell activation and inhibited the production of inflammation mediators in rats with ICH.

### USP11 stabilizes p53 by deubiquitination and inhibits KLF2 expression in microglial cells

**Figure 3:**
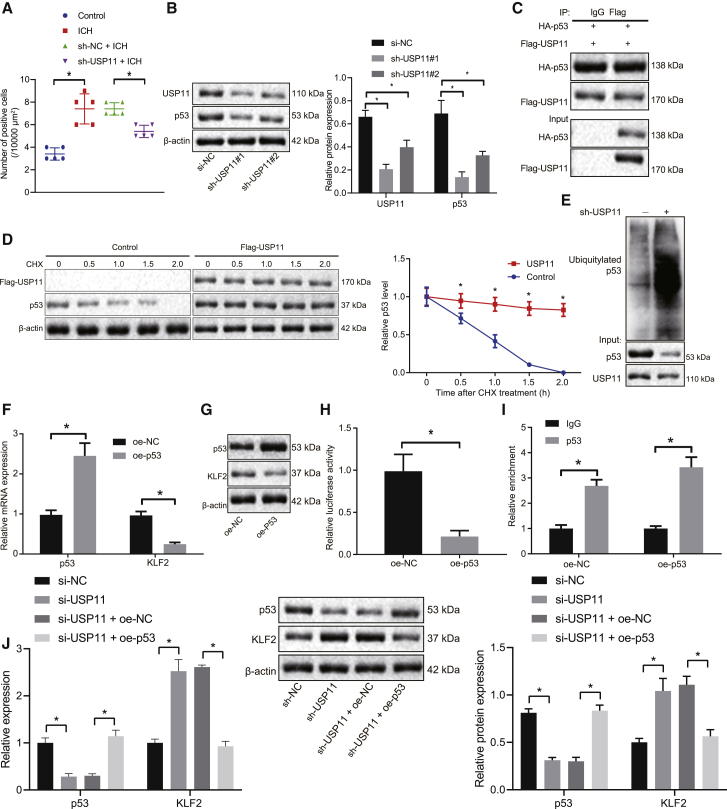
USP11 stabilizes p53 by deubiquitination and inhibits KLF2 transcription in microglial cells (A) Quantitative analysis of p53 expression in brain tissues of the rats after 72 h of ICH induction imaged by immunohistochemical staining. (B) USP11 and p53 protein expression in microglial cells infected with lentivirus expressing sh-USP11 as measured by western blot assay. (C) coIP showing interaction of USP11 and p53. (D) The stability of p53 treated with CHX in the presence of USP11; ∗p < 0.05 cells without infection. (E) Immunoprecipitation showing p53 ubiquitination level after USP11 overexpression. (F) qRT-PCR determination of p53 and KLF2 mRNA levels in the microglial cells infected with lentivirus expressing oe-p53. (G) Western blot assays of p53 and KLF2 protein levels in the microglial cells infected with lentivirus expressing oe-p53. (H) Luciferase activity of KLF2 promoter after p53 overexpression. (I) The enrichment of p53 in the KLF2 promoter region detected by ChIP. (J) Western blot and qRT-PCR assays to determine the protein and mRNA levels of p53 and KLF2 affected by USP11. ∗p < 0.05. Measurement data are presented as mean ± standard deviation. Comparisons among multiple groups were performed by one-way ANOVA, followed by Dunnett’s post hoc test. Statistical analysis in relation to time-based measurements within each group was realized using two-way ANOVA with Dunnett’s post hoc test. n = 5 in animal experiments, and all cell experiments were conducted independently in triplicate.

We then examined the speculation that USP11 may regulate nerve injury in ICH through the p53 pathway. As presented by the results of immunohistochemical staining, p53 expression was elevated in the brain tissues of the rats with ICH-like symptoms. USP11 silencing resulted in a reduction in p53 expression in the brain tissues of rats with ICH-like symptoms (Figure 3A). The protein levels of USP11 and p53 were quantified by western blot assay in microglial cells isolated from the rats with ICH-like symptoms. Infection with lentivirus-expressing short hairpin RNA (shRNA) sh-USP11 successfully reduced the USP11 protein expression and consequently downregulated the protein expression of p53 (Figure 3B). Co-immunoprecipitation (coIP) was performed to verify the interaction between USP11 and p53, and the results demonstrated the binding of USP11 to p53 (Figure 3C). To validate the effect of USP11 on p53 stability, we treated microglial cells with cycloheximide (CHX) in the presence of USP11. As expected, USP11 overexpression indeed inhibited the degradation of p53 and hence stabilized p53 expression (Figure 3D). Meanwhile, it was evidenced by immunoprecipitation that silencing USP11 boosted the p53 ubiquitination level (Figure 3E). To summarize, USP11 could stabilize p53 via deubiquitination.

Next, we investigated KLF2 as a potential downstream effector of p53. p53 was then overexpressed in microglial cells, which was validated by quantitative reverse-transcription polymerase chain reaction (qRT-PCR) and western blot assays. Accordingly, KLF2 expression was reduced by p53 overexpression (Figure 3F). Next, the dual-luciferase reporter gene assay and chromatin immunoprecipitation (ChIP) assay were conducted to identify the binding of p53 to the KLF2 promoter. By constructing the luciferase plasmid containing the KLF2 promoter sequence, the dual-luciferase reporter system exhibited that p53 overexpression considerably repressed the luciferase activity of the KLF2 promoter (Figures 3G and 3H). In the meantime, the enrichment of p53 in the KLF2 promoter region was enhanced after p53 overexpression (Figure 3I). This regulatory effect on KLF2 was further confirmed at protein and mRNA levels. Silencing USP11 elevated KLF2 protein and mRNA levels, whereas restoration of p53 abrogated the effect of sh-USP11 on KLF2 expression (Figure 3J). The above results convinced us that USP11 could deubiquitinate and stabilize p53 in microglial cells to repress KLF2 transcription.

### KLF2 overexpression inhibits activation of the NF-κB pathway to protect against ICH-induced inflammation

**Figure 4:**
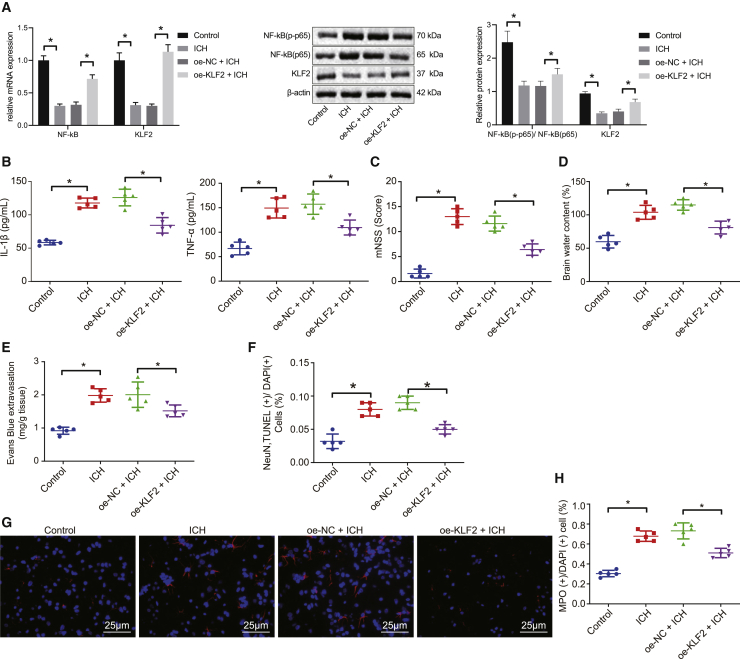
KLF2 overexpression inhibits NF-κB pathway activation and protects rats from ICH-caused nerve injury and inflammation damage (A) Western blot and qRT-PCR assays for measurement of KLF2, NF-κB (p-p65), and NF-κB (p65) protein and mRNA levels in the rat brain tissues. (B) ELISA detection of TNF-α and IL-1β expression in the rat brain tissues. (C) mNSS value in the rats with ICH-like symptoms in the presence of KLF2. (D) Determination of brain water content in the rats with ICH-like symptoms in the presence of KLF2. (E) Evans blue staining showing BBB permeability in the rats with ICH-like symptoms in the presence of KLF2. (F) Quantitative analysis of neuron apoptosis by TUNEL and NeuN staining in the rats with ICH-like symptoms in the presence of KLF2. (G) Immunofluorescence images for evaluating microglial cell activation in the rats with ICH-like symptoms in the presence of KLF2 (original magnification 400×). (H) Quantitative analysis of neutrophil infiltration after immunofluorescence staining in the brain tissues of the rats with ICH-like symptoms in the presence of KLF2. ∗p < 0.05. Measurement data are presented as mean ± standard deviation. Comparisons among multiple groups were performed by one-way ANOVA, followed by Dunnett’s post hoc test. n = 5.

Subsequently, we explored the effects of KLF2 on NF-κB pathway activation and the involvement of KLF2 and NF-κB in ICH-triggered inflammation. KLF2 was overexpressed in the rats with ICH-like symptoms by lentivirus infection. The protein and mRNA levels of KLF2 and NF-κB (p65) in the brain tissues were measured by western blot and qRT-PCR assays after lentivirus infection. Compared with the sham-operated rats, KLF2 protein and mRNA levels were diminished, whereas NF-κB (p65) protein level and its phosphorylation levels were raised in the rats with ICH-like symptoms. When KLF2 was overexpressed, the NF-κB phosphorylation level was suppressed (Figure 4A). Besides, overexpression of KLF2 considerably reduced the contents of TNF-α and IL-1β in the brain tissues of rats with ICH, ascertained by ELISA (Figure 4B). Furthermore, mNSS value (Figure 4C) and the brain water content (Figure 4D) of the rats with ICH-like symptoms were decreased by KLF2 overexpression. Besides, upregulation of KLF2 reduced BBB permeability and suppressed the neuron apoptosis in the brain tissues of the rats with ICH-like symptoms, as shown by Evans blue staining (Figure 4E) and TUNEL-NeuN dual staining (Figure 4F). Finally, mitigated microglial cell activation and neutrophil infiltration in the brain tissues of rats with ICH-like symptoms were observed in response to KLF2 overexpression (Figures 4G and 4H). To briefly conclude, KLF2 overexpression inhibits NF-κB activation to prevent ICH-caused nerve injury and inflammation damage.

### USP11 aggravates neurological impairment and inflammation in the rats with ICH-like symptoms through p53-induced repression of KLF2

**Figure 5:**
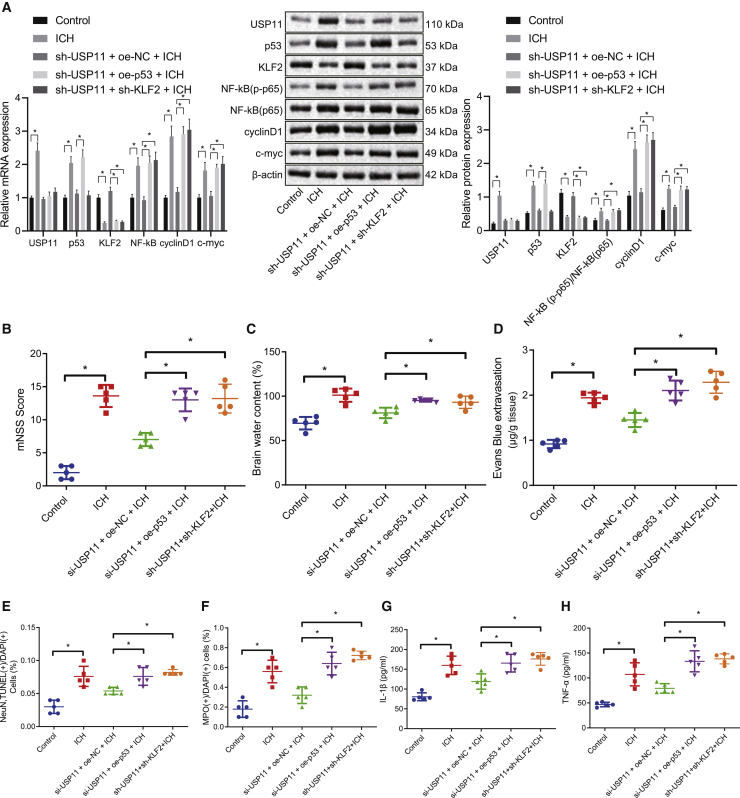
USP11 inhibits KLF2 via p53 and activates the NF-κB pathway to deteriorate neurological impairment and inflammation in rats with ICH-like symptoms (A) Western blot and qRT-PCR assays for the protein and mRNA levels of USP11, p53, KLF2, NF-κB (p-p65), NF-κB (p65), and downstream markers of the NF-κB pathway (cyclinD1, c-*myc*) in the brain tissues of rats with ICH-like symptoms. (B) mNSS of rats with ICH-like symptoms. (C) Brain water content in rats with ICH-like symptoms determined by wet-dry weight method. (D) BBB permeability in the brain tissues of the rats with ICH-like symptoms determined by Evans blue staining. (E) Quantitative analysis of neuron apoptosis by TUNEL and NeuN staining in the brain tissues of the rats with ICH-like symptoms. (F) Quantitative analysis of microglial cell activation by immunofluorescence staining. (G) Neutrophil infiltration in the brain tissues of the rats with ICH-like symptoms. (H) ELISA quantification of TNF-α and IL-1β expression in the brain tissues of the rats with ICH-like symptoms. ∗p < 0.05. Measurement data are presented as mean ± standard deviation. Comparisons among multiple groups were performed by one-way ANOVA, followed by Dunnett’s post hoc test. n = 5.

Following the aforementioned *in vitro* experiments, we then explored the *in vivo* effects of USP11 and p53-mediated KLF2 on the progression of ICH. As reflected by western blot and qRT-PCR assays, USP11 knockdown led to upregulated expression of KLF2, as well as downregulated levels of NF-κB (p65), its phosphorylation (p-p65), and downstream markers of the NF-κB pathway (cyclinD1, c-*myc*) in the brain tissues of rats with ICH-like symptoms; these effects of USP11 knockdown alone could be reversed by its combination with either p53 overexpression or KLF2 knockdown (Figure 5A). Also, the combination of USP11 knockdown and restoration of p53 or KLF2 knockdown elevated the mNSS value (Figure 5B) and brain water content (Figure 5C) reduced by USP11 knockdown alone in rats with ICH-like symptoms. Meanwhile, BBB permeability suppressed by USP11 silencing alone was increased when it was combined with p53 restoration or KLF2 silencing (Figure 5D). Additional restoration of p53 or knockdown of KLF2 also reinforced the neuron apoptosis (Figure 5E), microglial cell activation, and neutrophil infiltration (Figures 5F and 5G) in the brain tissues that had been suppressed by USP11 knockdown. Further, p53 overexpression or KLF2 knockdown reversed the inhibitory effects of USP11 silencing on levels of IL-1β and TNF-α in the brain tissues of rats with ICH-like symptoms (Figure 5H). Collectively, USP11 inhibits KLF2 via upregulating p53, thus facilitating the ICH-induced neurological impairment and inflammation.

## Discussion

ICH is a life-threatening illness with high mortality, few effective therapies, and poor prognosis.^2^ Accordingly, the focus of ICH research has shifted from emergency interventions to post-hemorrhage management, where one of the main tasks is to control the ICH-induced inflammatory response.^32^, ^33^, ^34^ To develop novel targets for the management of ICH, we focused on the implications of USP11 in the pathology of post-ICH inflammatory response. In the present study, we elucidated the regulatory role of the USP11-mediated p53/KLF2/NF-κB axis in ICH-induced inflammation and suggested USP11 as a potential anti-inflammatory target for ICH treatment. Also, our results demonstrated the neuroprotective effect of silencing USP11 supported by its contribution to protection against neuron apoptosis and neurological impairment.

In our research, USP11 level was increased after ICH, while downregulation of USP11 was found to protect the rats from post-ICH neuron apoptosis, which is consistent with a previous report.^8^ It has also been established that increased BBB permeability could result in acute microglial cell activation and excessive inflammation,^35^ which contribute to brain injury after ICH.^36^ The *in vivo* data of this study further substantiated that USP11 knockdown resulted in reductions in mNSS value, brain water content, and BBB permeability, as well as suppression of microglial cell activation, suggestive of the neuroprotective significance of silencing USP11. In addition to those effects, USP11 knockdown also repressed neutrophil infiltration and the production of proinflammatory cytokines IL-1β and TNF-α, indicative of an anti-inflammatory potency of silencing USP11. Intriguingly, apart from USP11, other deubiquitinating enzymes, such as TNAIP3^37^ OTUB1,^38^ and USP4,^39^ are also dysregulated after ICH and play either protective or injurious roles. Herein, we consider that deubiquitination is a critical pathological process in ICH. Agonists or antagonists of these deubiquitinases may serve as an innovative strategy for the management of ICH.

USP11 can bind to varied substrates and modulate their stabilization and deubiquitination. For instance, USP11 can reverse the ubiquitination and degradation of p21, a cyclin-dependent kinase inhibitor, and hence mediate the DNA damage response and apoptosis.^40^ The present study identified that USP11 could stabilize p53 through deubiquitination, although this positive regulation has also been reported by Ke et al.^18^ in response to DNA damage. This study further suggested their interaction in the central nervous system. The data provided evidence that USP11 positively regulated p53 expression to aggravate the ICH-induced neurological impairment and inflammation. p53 is a key inducer of programmed cell death, while blocking the activation of p53 contributes to suppressing neuroapoptosis and neuroinflammation and relieving neurological and cognitive dysfunction. ^41^^,^^42^

The p53-mediated suppression of KLF2 by binding to its promoter has been reported in endothelial cells^21^ A 27-bp sequence in the corn 221-bp KLF2 promoter has been identified to be essential for p53-induced repression of KLF2 transcription. Although we did not investigate the p53 response element in this study, we experimentally determined the binding of p53 to KLF2 promoter by dual-luciferase reporter gene and ChIP assays. KLF2 is a hub gene that could interact with transcriptional master regulator NF-κB, which can induce inflammation through controlling the transcription of several proinflammatory cytokines, such as IL-1β and TNF-α.^43^ Our findings verified that KLF2 activated NF-κB to restrain ICH-induced inflammation and neurological dysfunction. Accumulating evidence has documented the role of NF-κB as a key regulator of inflammatory signals.^44^ Also, NF-κB activation is closely associated with the cell apoptosis in the perihematomal brain tissues caused by ICH.^45^ Besides, NF-κB pathway activation by endogenous lactate activation can promote angiogenesis and neurogenesis after ICH, suggesting the importance of the NF-κB pathway in angiogenesis and neurogenesis post-ICH.^46^ Sun et al.^9^ have reported that USP11 catalyzes deubiquitination of IκBα and restrains its degradation to inhibit NF-κB activation.

**Figure 6:**
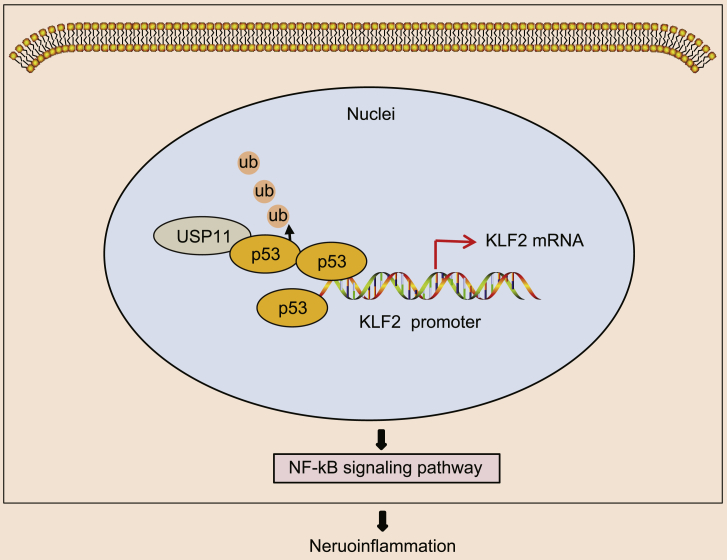
Schematic diagram of USP11 in ICH via p53/KLF2/NF-κB USP11 induces deubiquitination of p53 to enhance the stability of p53, which binds to the KLF2 promoter, whereby activating the NF-κB pathway and ICH-induced neurological impairment and inflammation.

As summarized in Figure 6, the involvement of the p53/KLF2/NF-κB axis in neuroinflammation has been established by our study as the molecular mechanism of the pro-inflammatory gene USP11. Deubiquitinase USP11 stabilized p53, which binds to the KLF2 promoter and represses KLF2 transcription, leading to activation of the NF-κB pathway. The NF-κB complex then activates microglia and neutrophils, and enhances the release of pro-inflammatory mediators, resulting in post-ICH inflammation. Those findings aid in the understanding of anti-inflammatory and neuroprotective mechanisms and highlight targeting USP11 as an anti-inflammatory strategy for ICH treatment.

## Materials and methods

### Ethics statement

The animal study was approved by the Animal Care and Use Committee of Tangshan People’s Hospital and conducted following the National Institutes of Health guidelines.

### Establishment of a rat model of ICH and animal treatment

A total of 60 adult male Sprague-Dawley rats with the body weight of 280–300 g were purchased from Hebei Provincial Medical Animal Experiment Center. Rats were housed at a constant ambient temperature (22°C ± 1°C) during a 12-h/12-h light-dark cycle. Collagenase, as a metalloproteinase, can decompose the collagen on the intercellular matrix and the basement membrane of blood vessels. It can damage the collagen on the basement membrane of cerebral blood vessels and destroy the BBB, which induces blood oozing afterward, and the accumulated blood gradually fuses into sheet-like hemorrhage after about 4 h. The size of the bleeding area depends on the injected amount of collagenase. Subsequently, rats were anesthetized by intraperitoneal injection with 3% pentobarbital sodium (50 mg/kg) and fixed on a three-dimensional positioning frame. A 29G needle was inserted 0.5 mm anterior, 3.5 mm lateral, and 5.5 mm in depth to deliver type IV bacterial collagenase (2.0 μL, 0.5 U; Sigma, St. Louis, MO, USA) into the right hemisphere. The needle was left in place for 10 min after injection. The temperature was maintained at 37°C ± 0.5°C during the entire period. The sham-operated rats (n = 5) only underwent needle insertion without injection of the type IV collagenase. Rats that developed ICH-like symptoms were infected with lentivirus-expressing shRNA negative control (sh-NC), shRNA against USP11 (sh-USP11), NC overexpression plasmid (oe-NC), KLF2 overexpression plasmid (oe-KLF2), sh-USP11, and oe-NC in combination, or sh-USP11 and oe-p53 in combination, respectively, with five rats for each treatment.

### Cell isolation and culture

Human embryonic kidney cell line HEK293T purchased from American Type Culture Collection (ATCC; CRL-1573; Manassas, VA, USA) was cultured in Dulbecco’s modified Eagle’s medium (DMEM) supplemented with 10% (v/v) fetal bovine serum (FBS) in the incubator (37°C, 5% CO_2_). Microglial cells were derived from the cerebral cortex of neonatal rats who aged 1–2 days. The cell differentiation of neonatal rats was low, and it was easier to culture microglial cells successfully *in vitro*. Microglial cells were co-cultured with glial for 10 days, and then microglia were isolated by shaking and cultured separately. Microglial cells were isolated from Sprague-Dawley (SD) rats. In detail, the isolated microglial cells were cultured in a 75-mL flask in DMEM/F12 medium (GIBCO, Carlsbad, CA, USA) supplemented with 10% (v/v) fetal calf serum (FCS; HyClone Laboratories, Logan, UT, USA), penicillin (100 U/mL), and streptomycin (100 mg/mL) for 14 days. Then microglial cells were harvested from the primary mixed glial cell culture solution by shaking on the 10th day. The flask was placed on a rotary oscillator (SC400; Dam Industry, Shanghai, P.R. China) at 37°C, 300 rpm overnight. Microglial cells were used for the following experiments after another 2 days of culturing.

### Lentivirus preparation and infection

The overexpression plasmids and sh-RNAs were packaged using the lentiviral vectors: LV5-GFP overexpression vector and pSIH1-H1-copGFP shRNA vector, respectively. sh-USP11 was synthesized by GenePharma (Shanghai, P.R. China). The packaged lentiviruses were transduced into HEK293T cells. The supernatant was collected after being cultured for 48 h, filtered, and centrifuged. The virus titer in the harvested supernatant was detected. Microglial cells were infected with the following lentivirus: sh-NC, sh-USP11, oe-NC, sh-USP11 + oe-NC, sh-USP11 + oe-p53. Afterward, the microglial cells were trypsinized and pipetted to a cell suspension at a concentration of 5 × 10^4^ cells/mL. The suspension was placed in a six-well plate (2 mL/well) and incubated at 37°C overnight. The following experiments were conducted after 48 h of transduction. Virus infection *in vivo* was performed 4 weeks before the establishment of the ICH model. Rats were intraperitoneally anesthetized with 3% pentobarbital sodium (50 mg/kg) and placed on a stereotaxic frame. Then, PBS-dissolved lentiviral particles at a titer of 5 × 10^12^ were injected into the right cerebral hemisphere of rats 0.5 mm in front of the forearm 10 mm, 3.5 mm outside the midline, and 5.5 mm deep using a 29-gauge needle. After injection, the needle was fixed in that position for 10 min. Throughout the process, the core temperature was maintained at 36.5°C–37.5°C. The control group was injected with a control virus that did not silence or overexpress the target gene.

### mNSS test

Neurologic deficit was scored by evaluating abnormal movements and motor, sensory, and reflex deficits at 72 h after ICH induction using the 18-point neurological deficit scale in the mNSS system. The mNSS test consists of movement, sensory, balance, and reflex tests. The total score is 18 points, and the higher the score, the more severe the neurological damage. It is widely used to assess the degree of nerve injury caused by cerebral hemorrhage. Therefore, the test was operated by three people, the injury was scored in a triple-blind manner, and the scores were averaged. After the ICH model was constructed, if in the first round of scoring animals were found to have symptoms such as difficulty breathing, premature death, or excessive bleeding during surgery, they were all excluded and the alternate rats would be used in subsequent experiments.

### Wet-dry weight method

Brain edema was assessed using the wet-dry weight method. In brief, rats were euthanized on day 7 after ICH. The brains were excised and hemorrhage hemispheres, contralateral hemispheres, and cerebellums were separated immediately and weighted in an electronic analytical balance (Sartorius BS 210 S, Gottingen, Germany), which was recorded as the wet weight (WW). The cerebellum was used as internal control. Those tissues were then dried at 110°C for 24 h, and dry weight (DW) was obtained. The brain water content was calculated using the following formula: (WW − DW)/(WW) × 100%.

### Evans blue staining assay

The BBB permeability was assessed by the extravasation of Evans blue dye (Wako Pure Chemical Corporation, Osaka, Japan). Rats were anesthetized and injected with Evans blue solution (2% in saline, 4 mL/kg) from the femoral vein. After 2 h, rats were re-anesthetized and perfused with PBS buffer (250 mL, 0.01 M, pH 7.4) to clear the Evans blue in the cerebral circulation. Brain hemispheres were quickly removed and separated into the left and right hemispheres. The samples were incubated in trichloroacetic acid (50%, 2 mL), homogenized, and centrifuged at 15,000 rpm for 20 min. The supernatant (1 mL) was diluted with ethanol (3 mL). The fluorescence was detected on a microplate reader with the excitation wavelength of 620 nm and the emission wavelength of 680 nm. The results were presented as Evans blue dye leakage (mg)/brain weight (g).

### Immunohistochemical staining

First, the paraffin-embedded brain tissues were sliced into 4-μm-thick sections, which were then dewaxed in xylene and rehydrated in ethanol and deionized water. The paraffin-embedded brain sections were heated in a microwave oven for 20 min in ethylenediaminetetraacetic acid (EDTA) buffer (50 mM Tris, 1 mM EDTA, pH 8.5) for antigen retrieval. Endogenous peroxidase activity was inactivated using 0.3% H_2_O_2_ for 10 min, and the sections were washed by PBS buffer. The sections were blocked by 5% bovine serum albumin (BSA) for 20 min. The slides were incubated overnight at 4°C with the primary antibodies: anti-USP11 antibody (anti-mouse, 1:100; Santa Cruz Biotechnology, Dallas, TX, USA). After PBS washing, the section was incubated with biotinylated goat anti-rat IgG secondary antibody for 20 min and then incubated with horseradish peroxidase (HRP)-streptavidin reagent (Innova Biosciences, Montlucon, France) for 20 min. Finally, the sections were developed by 3,3-diaminobenzidine (DAB) and counter-stained with hematoxylin. Images were captured under a microscope (Leica-DM2500; Leica, Wetzlar, Germany). The number of immunopositive cells in the intracerebral hematoma region was counted in a blinded manner and expressed as number per 0.1-mm^2^ areas.

### Immunofluorescence and TUNEL staining

The brain sections were rinsed with PBS buffer and blocked in 10% BSA for 1 h. The sections were then incubated at 4°C for 12–16 h with rabbit anti-USP11 (1:200; Alomone Lab, Jerusalem, Israel), rabbit anti-Iba-1 (1:200; Wako, Osaka, Japan), and rat anti-myeloperoxidase (anti-MPO; 1:100; Santa Cruz Biotechnology, Dallas, TX, USA). After PBS washing, the sections were incubated at 25°C for 1 h with Alexa Fluor 647- or Alexa Fluor 488-conjugated donkey anti-rat IgG (Thermo Fisher, Waltham, MA, USA) and Cy3-conjugated donkey anti-rabbit IgG (Jackson Immuno-Research, West Grove, PA, USA). The sections were re-stained by 4′6-diamidino-2-phenylindole (DAPI; 10 μg/mL; Sigma) at 25°C for 15 min. To determine the neuron apoptosis, we strained the sections with TUNEL (Cell Death Detection Kit; Roche, Basel, Switzerland) and rabbit anti-NeuN (1:200; Abcam, Cambridge, UK). All the sections were observed in a blind manner under an Olympus BX51 fluorescent microscope (Olympus, Tokyo, Japan) or a laser scanning confocal microscope (FV500; Olympus, Tokyo, Japan). Five fields of the edge of intracerebral hematoma region per section and four sections per animal (n = 5) were selected. The MPO^+^ cells were counted in the fields. The total number of TUNEL- and NeuN-positive cells in the five fields near the injury area was counted using ImageJ software (National Institute of Health, Bethesda, MD, USA).

### ELISA

On day 3 after ICH induction, the intracerebral hematoma tissues (n = 5) were homogenized and centrifuged. The supernatants were collected, and the levels of inflammatory cytokines (pg/mg), TNF-α, and IL-1β were detected using ELISA reagent (Dakewe Biotech, Shenzhen, Guangdong, P.R. China) according to the manufacturer’s instructions.

### RNA extraction and quantitation assay

Microglial cells were lysed, and total RNAs were isolated from cells and tissues using a TRIzol Plus RNA Purification Kit (Invitrogen, Carlsbad, CA, USA). Quality and concentration of RNA were verified on an ND-1000 UV-Vis spectrometer (NanoDrop, Wilmington, DE, USA). mRNA was reversely transcribed to cDNA using PrimeScript RT Reagent Kit with gDNA Eraser (RR047A; Takara, Kusatsu, Japan) in a total volume of 20 μL system. The real-time qPCR was conducted using a SYBR Premix Ex Taq II (Perfect Real Time) kit (DRR081; Takara, Kusatsu, Japan). The reaction was conducted in a real-time PCR instrument (ABI7500; ABI, Foster City, CA, USA) with GAPDH as the internal reference. Primer sequences for qRT-PCR are listed in Table 1. The 2^−ΔΔCT^ method was applied to calculate the relative expression of genes with the formula: ΔΔCT = ΔCt _experiment group_ − ΔCt _control group_, where ΔCt = Ct _target gene_ − Ct _GAPDH_.

**Table 1:** Primer sequences for qRT-PCR

Gene of interest	Sequence
USP11	Forward: 5′-CCCAGTTGCGCCAGTATCATT-3′
	Reverse: 5′-TGATACTGGCGCCATTGTGTT-3′
p53	Forward: 5′-ACCTATGGAAACTACTTCCTGAAA-3′
	Reverse: 5′-CTGGCATTCTGGGAGCTTCA-3′
KLF2	Forward: 5′-GCCTATCTTGCCGTCCTTT-3′
	Reverse: 5′-AGTCCAGCACGCTGTTTAG-3′
GAPDH	Forward: 5′-GGGAAACCCATCACCATCTT-3′
	Reverse: 5′-CCAGTAGACTCCACGACATA-3′

GAPDH, glyceraldehyde-3-phosphate dehydrogenase; KLF2; Kruppel-like factor 2; qRT-PCR, quantitative reverse-transcription polymerase chain reaction; USP11, ubiquitin-specific protease 11.

### Western blot assay

Total proteins were extracted from cells and tissues using radioimmunoprecipitation assay (RIPA) lysis buffer (C0481; Sigma, Shanghai, P.R. China) at 4°C for 15 min, followed by centrifugation at 15,000 rpm for 15 min according to the user manual. The supernatant was collected, and the protein concentration was measured and normalized using a bicinchoninic acid (BCA) kit (23227; Thermo Fisher). Proteins were separated by sodium dodecyl sulfate-polyacrylamide gel electrophoresis (SDS-PAGE) and transferred onto polyvinylidene fluoride (PVDF) membrane (Millipore, Billerica, MA, USA). The membrane was blocked in 5% BSA for 1 h and then incubated at 4°C overnight with diluted primary antibodies: rabbit anti-NF-κB (p-p65) (ab86299, 1:2,000; Abcam), rabbit anti-NF-κB (p65) (ab16502, 1:1,000; Abcam), rabbit anti-c-*myc* (ab32072, 1:1,000; Abcam), rabbit anti-cyclinD1 (ab16663, 1:1,000; Abcam), rabbit anti-USP11 (ab109232, 1:1,000; Abcam), rabbit anti-p53 (ab26, 1:1,000; Abcam), and rabbit anti-KLF2 (ab194486, 1:1,000; Abcam). After incubation, the membrane was washed with TBST buffer for 5 min three times. HRP-labeled anti-rabbit IgG H&L (ab205718, 1:10,000; Abcam) was added for another 1.5-h incubation at room temperature, followed by TBST washing for 5 min three times. The immunoblots were visualized with enhanced chemiluminescence (ECL) reagents (NCI4106; Pierce, Rockford, IL, USA). The images were captured and analyzed by ImageJ 1.48u (Bio-Rad, Hercules, CA, USA). β-Actin was served as the internal reference.

### *In vivo* ubiquitination assay

Hemagglutinin (HA)-p53, Myc-ubiquitin, and FLAG-USP11 were co-transfected into HEK293T cells for 36 h. The cells were then treated with MG132 (5 μM) for 3 h before collection. The harvested cells were lysed in RAPI buffer: SDS (0.5%, 5 g/L), sodium deoxycholate (0.5%, 5 g/L), Nonidet P-40 (0.5%), NaCl (150 mM), NaF (10 mM), β-glycerophosphate (20 mM), sodium orthovanadate (1 mM), phenylmethylsulfonyl fluoride (1 mM), leupeptin (10 μg/mL), and aprotinin (2 μg/mL). Cells were incubated with anti-HA at 4°C overnight and with protein G agarose beads for another 4 h. The immunoprecipitated proteins were boiled in SDS buffer and measured by western blot assay. All the antibodies used in this assay, including anti-FLAG (ab205606, 1:30), anti-β-actin (ab179467, 1:40), anti-HA (ab18181, 1:50), anti-Myc (ab32072, 1:20), anti-USP11 (ab109232, 1:50), and anti-p53 (ab26, 1:50), were purchased from Abcam.

### ChIP assay

ChIP assay was performed using the ChIP kit (Thermo Fisher, Waltham, MA, USA). The cells were fixed using 1% formaldehyde and sheared by sonication. The P65-DNA complex was immunoprecipitated using anti-p53 (ab131442, 1:50; Abcam) and filtered by protein G agarose beads. The KLF2-DNA crosslinking was reversed, and the DNA was purified and used as a template for qRT-PCR. Primers sequences for KLF2 promoters were as follows: forward, 5′-gcagtccgggctcccgcagtag-3′, and reverse, 5′-cttataggcgcggcaggcac-3′.

### Dual-luciferase reporter gene assay

The plasmid containing the KLF2 promoter sequence was constructed using the psiCHECK vector. Cells were plated at a density of 5 × 10^4^ cells per well in a 24-well plate and were transiently transfected using Lipofectamine 2000, and Renilla luciferase was taken as an internal control. Cells were harvested 24 h after transfection. Firefly and Renilla luciferase activities were measured using a dual-luciferase reporter assay kit.

### Statistical analysis

Statistical data were processed by SPSS 21.0 (IBM, Armonk, NY, USA). Measurement data were characterized as mean ± standard deviation. Comparisons among multiple groups were performed by one-way analysis of variance (ANOVA), followed by Dunnett’s post hoc test. Statistical analysis in relation to time-based measurements within each group was realized using two-way ANOVA with Dunnett’s post hoc test. A p value <0.05 indicated a significant difference.

### Data availability

The datasets generated and/or analyzed during the current study are available from the corresponding author on reasonable request.

## References

[1] Balami J.S., Buchan A.M. (2012). Complications of intracerebral haemorrhage. Lancet Neurol..

[2] Cordonnier C., Demchuk A., Ziai W., Anderson C.S. (2018). Intracerebral haemorrhage: current approaches to acute management. Lancet.

[3] Kreitzer N., Adeoye O. (2013). An update on surgical and medical management strategies for intracerebral hemorrhage. Semin. Neurol..

[4] Adeoye O., Broderick J.P. (2010). Advances in the management of intracerebral hemorrhage. Nat. Rev. Neurol..

[5] Lan X., Han X., Li Q., Yang Q.W., Wang J. (2017). Modulators of microglial activation and polarization after intracerebral haemorrhage. Nat. Rev. Neurol..

[6] Keep R.F., Hua Y., Xi G. (2012). Intracerebral haemorrhage: mechanisms of injury and therapeutic targets. Lancet Neurol..

[7] Ideguchi H., Ueda A., Tanaka M., Yang J., Tsuji T., Ohno S., Hagiwara E., Aoki A., Ishigatsubo Y. (2002). Structural and functional characterization of the USP11 deubiquitinating enzyme, which interacts with the RanGTP-associated protein RanBPM. Biochem. J..

[8] Xu Z., Li X., Chen J., Zhao J., Wang J., Ji Y., Shen Y., Han L., Shi J., Zhang D. (2016). USP11, Deubiquitinating Enzyme, Associated with Neuronal Apoptosis Following Intracerebral Hemorrhage. J. Mol. Neurosci..

[9] Sun W., Tan X., Shi Y., Xu G., Mao R., Gu X., Fan Y., Yu Y., Burlingame S., Zhang H. (2010). USP11 negatively regulates TNFalpha-induced NF-kappaB activation by targeting on IkappaBalpha. Cell. Signal..

[10] Ting X., Xia L., Yang J., He L., Si W., Shang Y., Sun L. (2019). USP11 acts as a histone deubiquitinase functioning in chromatin reorganization during DNA repair. Nucleic Acids Res..

[11] Lee E.W., Seong D., Seo J., Jeong M., Lee H.K., Song J. (2015). USP11-dependent selective cIAP2 deubiquitylation and stabilization determine sensitivity to Smac mimetics. Cell Death Differ..

[12] Surget S., Khoury M.P., Bourdon J.C. (2013). Uncovering the role of p53 splice variants in human malignancy: a clinical perspective. OncoTargets Ther..

[13] Chao C.C. (2015). Mechanisms of p53 degradation. Clin. Chim. Acta.

[14] Stevenson L.F., Sparks A., Allende-Vega N., Xirodimas D.P., Lane D.P., Saville M.K. (2007). The deubiquitinating enzyme USP2a regulates the p53 pathway by targeting Mdm2. EMBO J..

[15] Li M., Chen D., Shiloh A., Luo J., Nikolaev A.Y., Qin J., Gu W. (2002). Deubiquitination of p53 by HAUSP is an important pathway for p53 stabilization. Nature.

[16] Yuan J., Luo K., Zhang L., Cheville J.C., Lou Z. (2010). USP10 regulates p53 localization and stability by deubiquitinating p53. Cell.

[17] Yamaguchi T., Kimura J., Miki Y., Yoshida K. (2007). The deubiquitinating enzyme USP11 controls an IkappaB kinase alpha (IKKalpha)-p53 signaling pathway in response to tumor necrosis factor alpha (TNFalpha). J. Biol. Chem..

[18] Ke J.Y., Dai C.J., Wu W.L., Gao J.H., Xia A.J., Liu G.P., Lv K.S., Wu C.L. (2014). USP11 regulates p53 stability by deubiquitinating p53. J. Zhejiang Univ. Sci. B.

[19] Laptenko O., Prives C. (2006). Transcriptional regulation by p53: one protein, many possibilities. Cell Death Differ..

[20] Lu L., Huang W., Hu W., Jiang L., Li Y., Wu X., Yuan D., Li M. (2019). Kruppel-like factor 2 mediated anti-proliferative and anti-metastasis effects of simvastatin in p53 mutant colon cancer. Biochem. Biophys. Res. Commun..

[21] Kumar A., Kim C.S., Hoffman T.A., Naqvi A., Dericco J., Jung S.B., Lin Z., Jain M.K., Irani K. (2011). p53 impairs endothelial function by transcriptionally repressing Kruppel-Like Factor 2. Arterioscler. Thromb. Vasc. Biol..

[22] Zhou Y., Wang Y., Wang J., Anne Stetler R., Yang Q.W. (2014). Inflammation in intracerebral hemorrhage: from mechanisms to clinical translation. Prog. Neurobiol..

[23] Kim J.H., Kang S., Jung Y.N., Choi H.S. (2016). Cholecalciferol inhibits lipid accumulation by regulating early adipogenesis in cultured adipocytes and zebrafish. Biochem. Biophys. Res. Commun..

[24] Vinjamur D.S., Wade K.J., Mohamad S.F., Haar J.L., Sawyer S.T., Lloyd J.A. (2014). Krüppel-like transcription factors KLF1 and KLF2 have unique and coordinate roles in regulating embryonic erythroid precursor maturation. Haematologica.

[25] Li Q., Chen Y., Zhang X., Zuo S., Ge H., Chen Y., Liu X., Zhang J.H., Ruan H., Feng H. (2016). Scutellarin attenuates vasospasm through the Erk5-KLF2-eNOS pathway after subarachnoid hemorrhage in rats. J. Clin. Neurosci..

[26] Gilmore T.D. (2006). Introduction to NF-kappaB: players, pathways, perspectives. Oncogene.

[27] Nayak L., Goduni L., Takami Y., Sharma N., Kapil P., Jain M.K., Mahabeleshwar G.H. (2013). Kruppel-like factor 2 is a transcriptional regulator of chronic and acute inflammation. Am. J. Pathol..

[28] Das H., Kumar A., Lin Z., Patino W.D., Hwang P.M., Feinberg M.W., Majumder P.K., Jain M.K. (2006). Kruppel-like factor 2 (KLF2) regulates proinflammatory activation of monocytes. Proc. Natl. Acad. Sci. USA.

[29] Lingrel J.B., Pilcher-Roberts R., Basford J.E., Manoharan P., Neumann J., Konaniah E.S., Srinivasan R., Bogdanov V.Y., Hui D.Y. (2012). Myeloid-specific Krüppel-like factor 2 inactivation increases macrophage and neutrophil adhesion and promotes atherosclerosis. Circ. Res..

[30] Hickenbottom S.L., Grotta J.C., Strong R., Denner L.A., Aronowski J. (1999). Nuclear factor-kappaB and cell death after experimental intracerebral hemorrhage in rats. Stroke.

[31] Zeng J., Chen Y., Ding R., Feng L., Fu Z., Yang S. (2017). Isoliquiritigenin alleviates early brain injury after experimental intracerebral hemorrhage via suppressing ROS- and/or NF-kappaB-mediated NLRP3 inflammasome activation by promoting Nrf2 antioxidant pathway. J Neuroinflammation.

[32] Wagner K.R. (2007). Modeling intracerebral hemorrhage: glutamate, nuclear factor-kappa B signaling and cytokines. Stroke.

[33] Badjatia N., Monahan A., Carpenter A., Zimmerman J., Schmidt J.M., Claassen J., Connolly E.S., Mayer S.A., Karmally W., Seres D. (2015). Inflammation, negative nitrogen balance, and outcome after aneurysmal subarachnoid hemorrhage. Neurology.

[34] Wang J. (2010). Preclinical and clinical research on inflammation after intracerebral hemorrhage. Prog. Neurobiol..

[35] Ju F., Ran Y., Zhu L., Cheng X., Gao H., Xi X., Yang Z., Zhang S. (2018). Increased BBB Permeability Enhances Activation of Microglia and Exacerbates Loss of Dendritic Spines After Transient Global Cerebral Ischemia. Front. Cell. Neurosci..

[36] Wu J., Yang S., Xi G., Song S., Fu G., Keep R.F., Hua Y. (2008). Microglial activation and brain injury after intracerebral hemorrhage. Acta Neurochir. Suppl. (Wien).

[37] Lu J., Sun Z., Fang Y., Zheng J., Xu S., Xu W., Shi L., Mei S., Wu H., Liang F., Zhang J. (2019). Melatonin Suppresses Microglial Necroptosis by Regulating Deubiquitinating Enzyme A20 After Intracerebral Hemorrhage. Front. Immunol..

[38] Xie L., Li A., Shen J., Cao M., Ning X., Yuan D., Ji Y., Wang H., Ke K. (2016). OTUB1 attenuates neuronal apoptosis after intracerebral hemorrhage. Mol. Cell. Biochem..

[39] Liu C., Liu C., Liu H., Gong L., Tao T., Shen Y., Zhu S., Shen A. (2017). Increased Expression of Ubiquitin-Specific Protease 4 Participates in Neuronal Apoptosis After Intracerebral Hemorrhage in Adult Rats. Cell. Mol. Neurobiol..

[40] Deng T., Yan G., Song X., Xie L., Zhou Y., Li J., Hu X., Li Z., Hu J., Zhang Y. (2018). Deubiquitylation and stabilization of p21 by USP11 is critical for cell-cycle progression and DNA damage responses. Proc. Natl. Acad. Sci. USA.

[41] Yang L.Y., Greig N.H., Huang Y.N., Hsieh T.H., Tweedie D., Yu Q.S., Hoffer B.J., Luo Y., Kao Y.C., Wang J.Y. (2016). Post-traumatic administration of the p53 inactivator pifithrin-α oxygen analogue reduces hippocampal neuronal loss and improves cognitive deficits after experimental traumatic brain injury. Neurobiol. Dis..

[42] Li X.Q., Yu Q., Chen F.S., Tan W.F., Zhang Z.L., Ma H. (2018). Inhibiting aberrant p53-PUMA feedback loop activation attenuates ischaemia reperfusion-induced neuroapoptosis and neuroinflammation in rats by downregulating caspase 3 and the NF-κB cytokine pathway. J. Neuroinflammation.

[43] Jha P., Das H. (2017). KLF2 in Regulation of NF-κB-Mediated Immune Cell Function and Inflammation. Int. J. Mol. Sci..

[44] Ben-Neriah Y., Karin M. (2011). Inflammation meets cancer, with NF-κB as the matchmaker. Nat. Immunol..

[45] Zhang Z., Liu Y., Huang Q., Su Y., Zhang Y., Wang G., Li F. (2014). NF-κB activation and cell death after intracerebral hemorrhage in patients. Neurol. Sci..

[46] Zhou J., Liu T., Guo H., Cui H., Li P., Feng D., Hu E., Huang Q., Yang A., Zhou J. (2018). Lactate potentiates angiogenesis and neurogenesis in experimental intracerebral hemorrhage. Exp. Mol. Med..

